# Supplementary material for: Catalytically potent and selective clusterzymes for modulation of neuroinflammation through single-atom substitutions
Source: Nat Commun. 2021 Jan 7;12:114. doi: 10.1038/s41467-020-20275-0 (PMC7791071; doi:10.1038/s41467-020-20275-0)
Supplement: Supplementary file 1 — Supplementary Information [file 41467_2020_20275_MOESM1_ESM.pdf]

Supplementary Information

Catalytically Potent and Selective Clusterzymes for Modulation of Neuroinflammation Through Single-Atom Substitutions

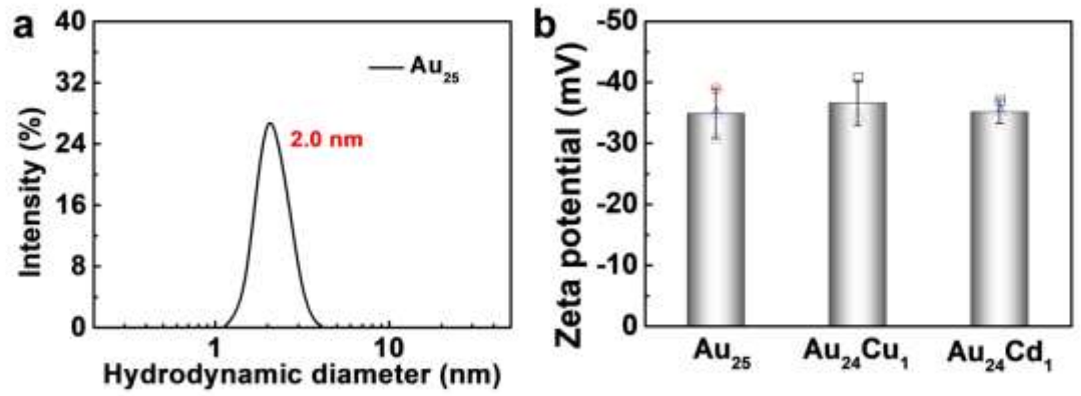

Supplementary Figure 1. a Hydrodynamic diameter of Au_{25} in PBS buffer, and the mean size is about 2.0 nm. **b** The zeta potentials of the clusterzymes indicated have good stability (n=3 independent experiments, data are presented as mean ± SD).

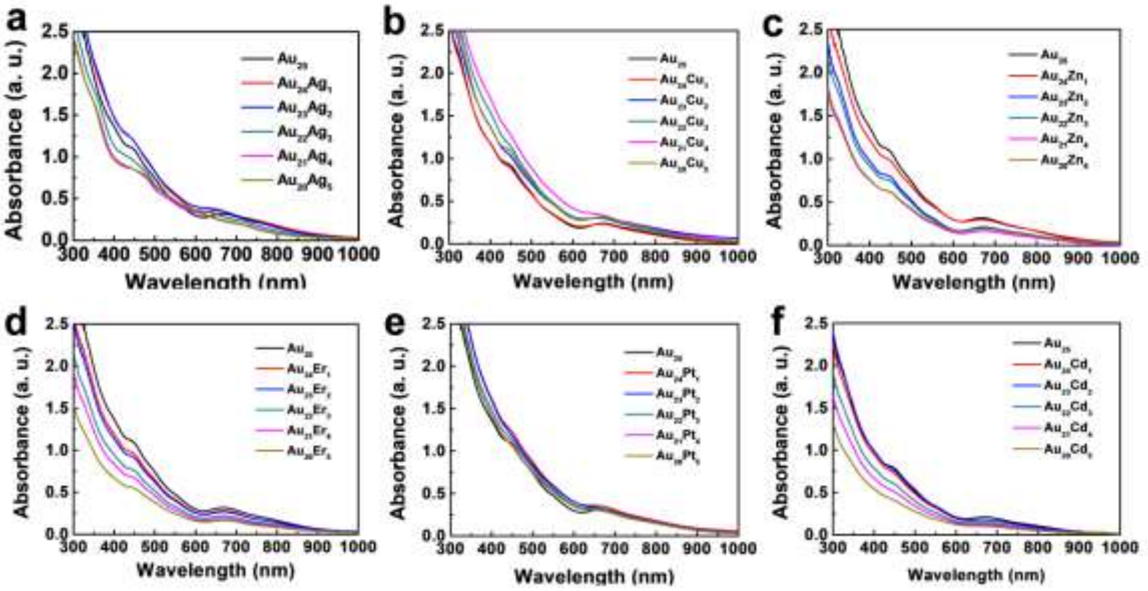

Supplementary Figure 2. Absorption spectra of Au_{25} substituted with different metals and different molar concentrations (Au:M=25:0, 24:1, 23:2, 22:3, 21:4, 20:5) **a** Ag, **b** Cu, **c** Zn, **d** Er, **e** Pt, **f** Cd.

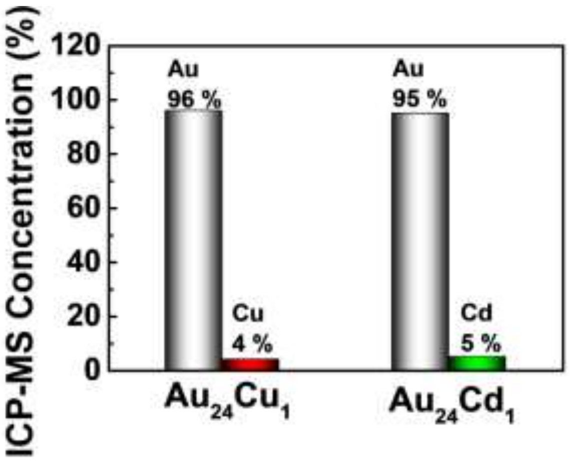

Supplementary Figure 3. The contents of metal elements in the clusterzymes were determined by ICP-MS method. Cu and Cd account for 4 % and 5 % of the total metal content, respectively, meaning that one Cu or Cd is substituted into Au_{25} skeleton.

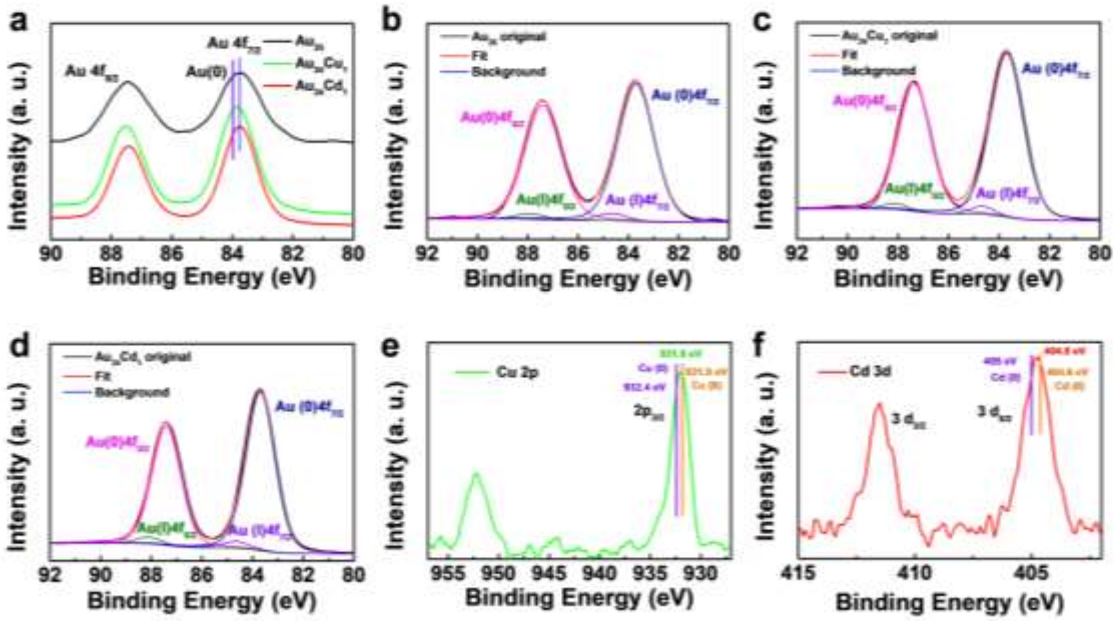

Supplementary Figure 4. X-ray photoelectron spectroscopy. **a** Au 4f region of Au_{25}, $Au_{24}Cu_1$ and $Au_{24}Cd_1$ clusterszymes. The high-resolution original and fitted Au 4f spectra of **b** Au_{25} and **c** $Au_{24}Cu_1$ **d** $Au_{24}Cd_1$ are shown. **e** Cu 2p XPS spectrum of $Au_{24}Cu_1$ and **f** Cd 3d spectrum of $Au_{24}Cd_1$. All spectra are obtained at the incident photon energy of 700 eV, and all binding energies were calibrated with a C 1s peak of 284.8 eV. Cu 2p peaks (Cu $2p_{3/2}$ = 931.8 eV) were observed in the reducing side relative to that of Cu(0) (id. = 932.4 eV). This indicates that partial charge transfer occurs from Au (electronegativity: 2.4) to Cu (electronegativity: 1.9). Cd 3d peaks (Cd $3d_{5/2}$ = 404.8 eV) were observed in the reducing side relative to that of Cd(0) (id. = 405 eV). This indicates that partial charge transfer occurs from Au (electronegativity: 2.4) to Cd (electronegativity: 1.69).

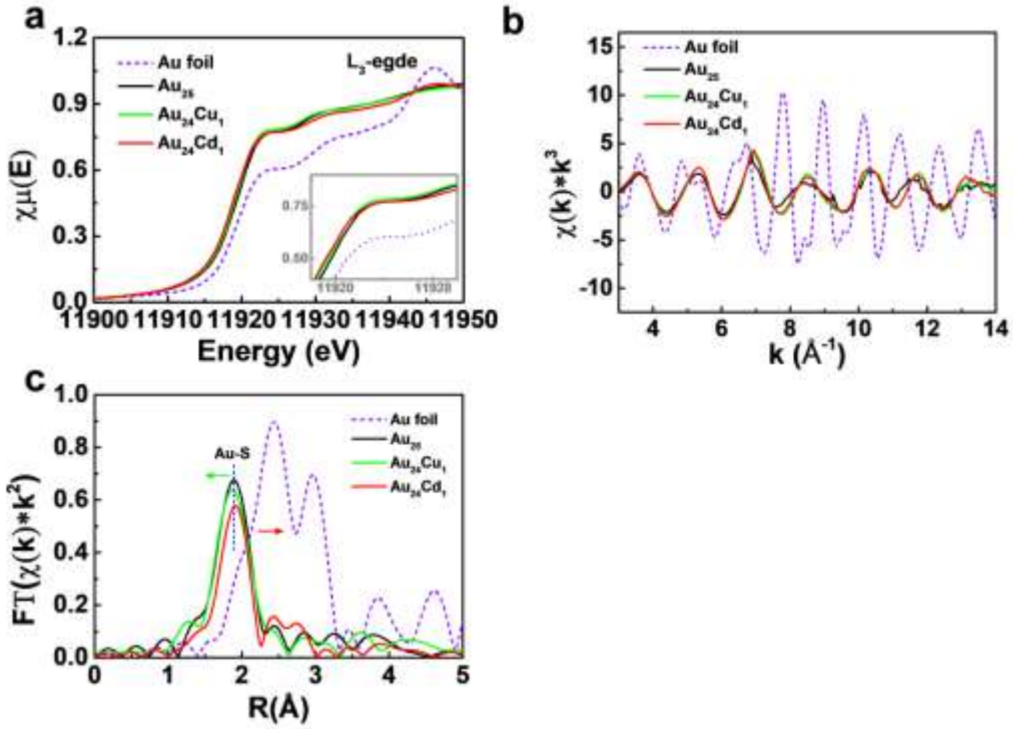

Supplementary Figure 5. a Au L_3-edge XANES of Au foil, Au_{25}, $Au_{24}Cu_1$ and $Au_{24}Cd_1$ clusterszymes, (inset, the region of white line). **b** Au L_3-edge FT-EXAFS spectra in R-space and **c** k-space of Au_{25} clusterzymes. Compared with Au foil, the XANES spectra of Au_{25}, $Au_{24}Cu_1$ and $Au_{24}Cd_1$ show higher energy edge absorption and significantly stronger white line transfer, indicating a significant contribution of Au(I) electron characteristics. FT-EXAFS spectrum shows that the Au-S of Au_{25} cluster is ~ 1.9 Å, which is not exist in Au foil. After substituting Cu and Cd, the Au-S still exists, indicating that Cu or Cd is substituted in the surface of Au_{13} core or oligomer site. The k-space of Au foil exists in the fcc oscillation patterns which are absent in Au_{25} clusters due to the small core size.

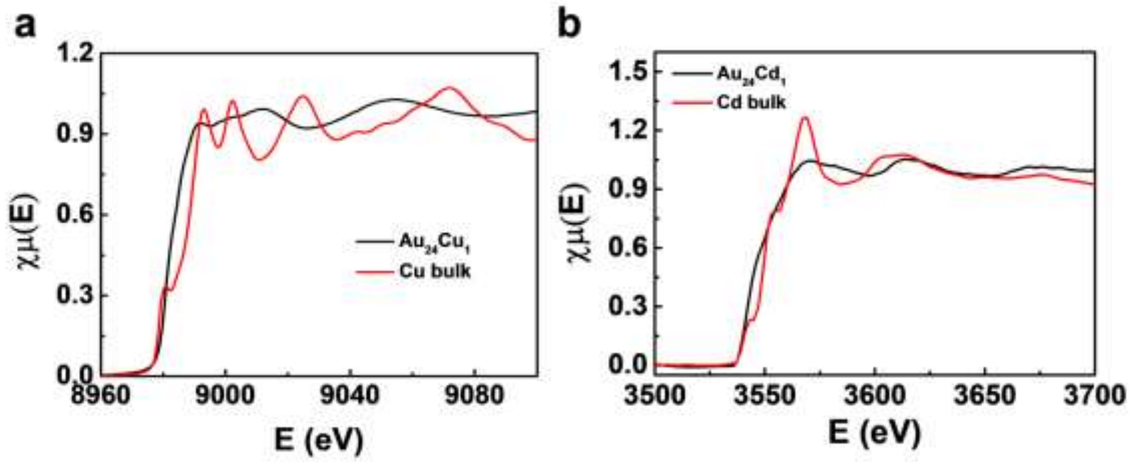

Supplementary Figure 6. a Cd L_3-edge and **b** Cu K-edge XANES spectra of $Au_{24}Cu_1$ and $Au_{24}Cd_1$ clusterszymes. It can be seen that the Cd-L_3 and Cu K-edge XANES spectra are different from the spectra of Cd and Cu bulk, showing characteristics of oxidized states of Cd and Cu.

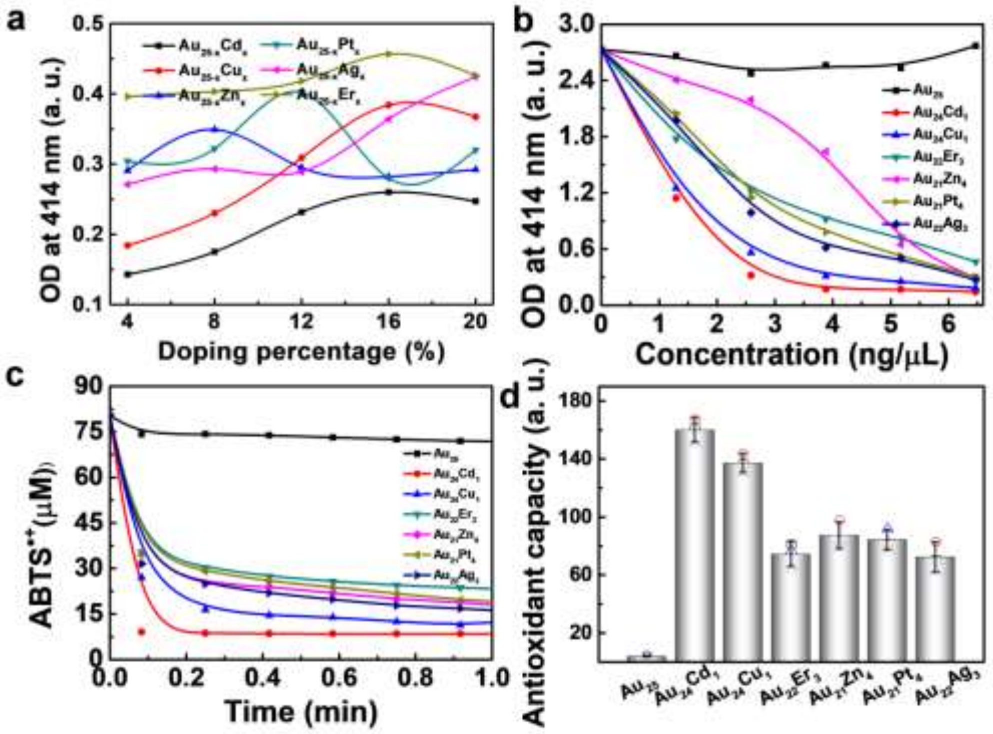

Supplementary Figure 7. a The total antioxidant capacity (T-AOC) of Au_{25} substituted with different metals (Cd, Cu, Zn, Pt, Ag, Er) and different concentrations (4-20 %). **b** The total antioxidant capacity of Au_{25} substituted with different metals varied with the increase of concentration (0-6.5 ng/ μL). **c** Time-dependent kinetics reaction of the total antioxidant capacity of Au_{25} substituted with different metals (5 ng/ μL). **d** Comparison of total antioxidant capacity of Au_{25} substituted with different metals (n=3 independent experiments, data are presented as mean ± SD).

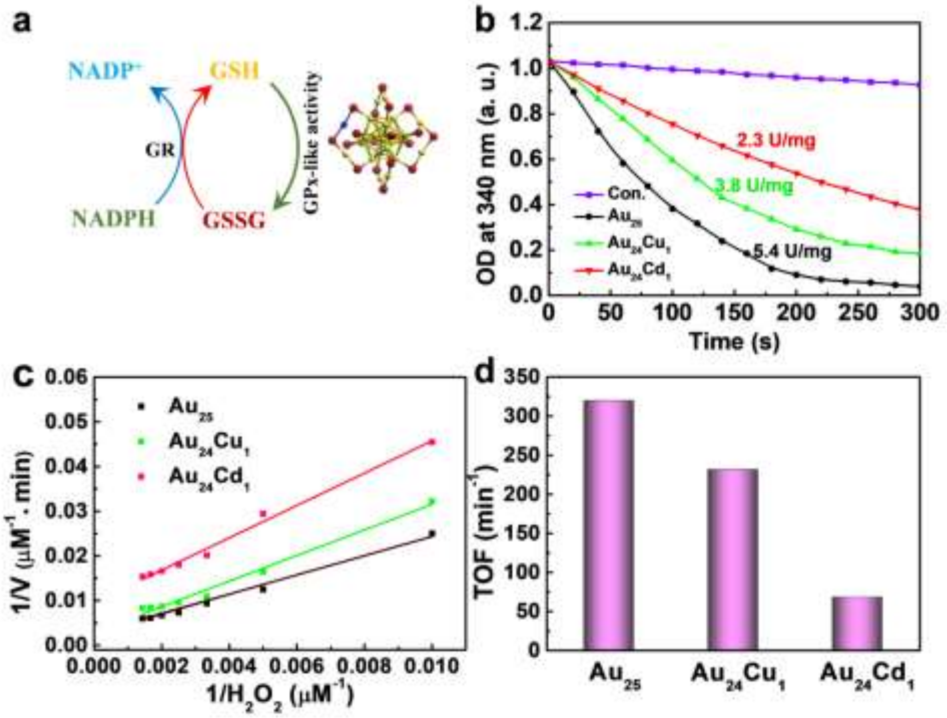

Supplementary Figure 8. a Schematic illustration of simulated glutathione peroxidase for the determination the activity of clusterzyme. **b** GPx-like activities with different clusterzymes (10 ng/μL). **c-d** The reaction kinetic analysis process of GPx-like activities. The reaction rate were determined with different concentrations of H_2O_2 as substrates in the presence of Au_{25}, $Au_{24}Cu_1$, $Au_{24}Cd_1$ clusterzymes (10 ng/μL). The V_{max} and K_m were calculated using the Lineweaver-Burk equation. The turnover frequency (TOF) = V_{max}/[E], E is the molality of clusterzymes.

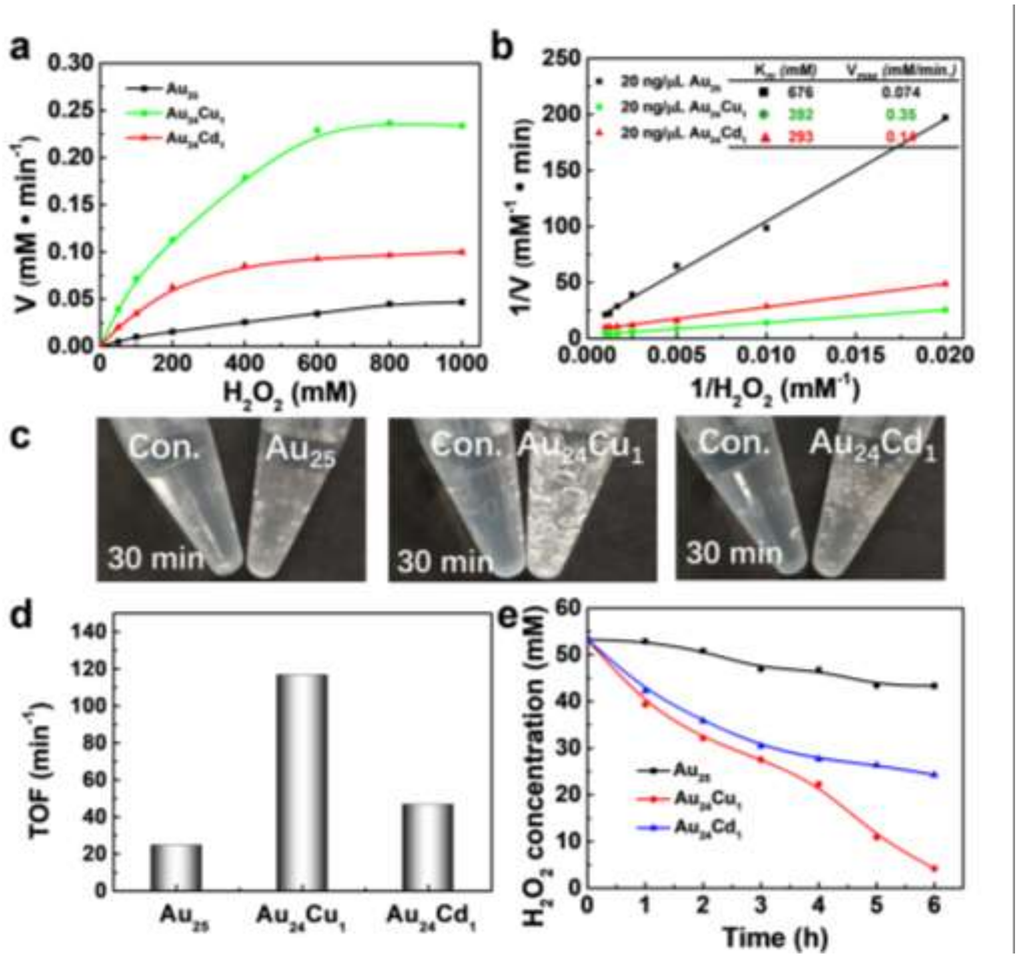

Supplementary Figure 9. a, b, d The reaction kinetic analysis process of CAT-like activity. The maximum reaction velocity (V_{max}) and Michaelis-Menten constant (K_m) were calculated using the Lineweaver-Burk equation. TOF = V_{max}/[E], and E is the molality of clusterzymes. **c** O_2 bubble formation. 200 μL, 10 M H_2O_2 was incubated with 50 ng/μL of different enzymes for 30 min. **e** Time-dependent absorbance at 240 nm of 200 μL, 53 μM H_2O_2 treated with 10 ng/μL different clusterzymes in neutral PBS.

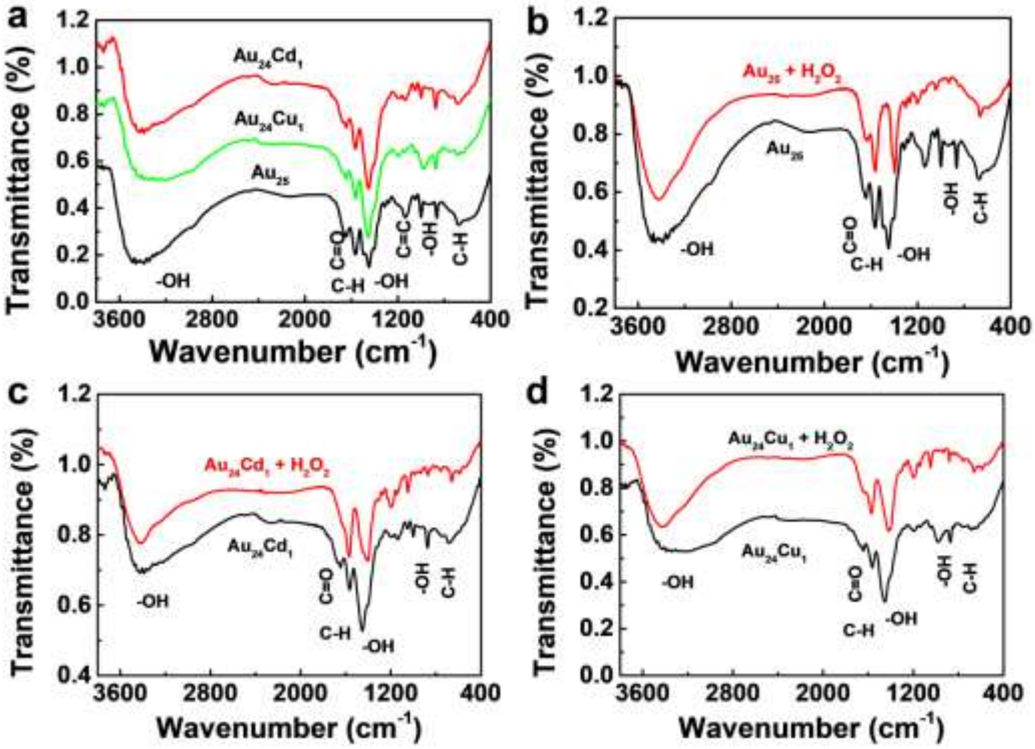

Supplementary Figure 10. FT-IR spectra of Au_{25}, $Au_{24}Cu_1$ and $Au_{24}Cd_1$ clusterzymes **a** before and **b-d** after the reaction of H_2O_2. The infrared vibration peak do not change obviously before and after the reaction of H_2O_2, which indicate that the ligand structure hardly change.

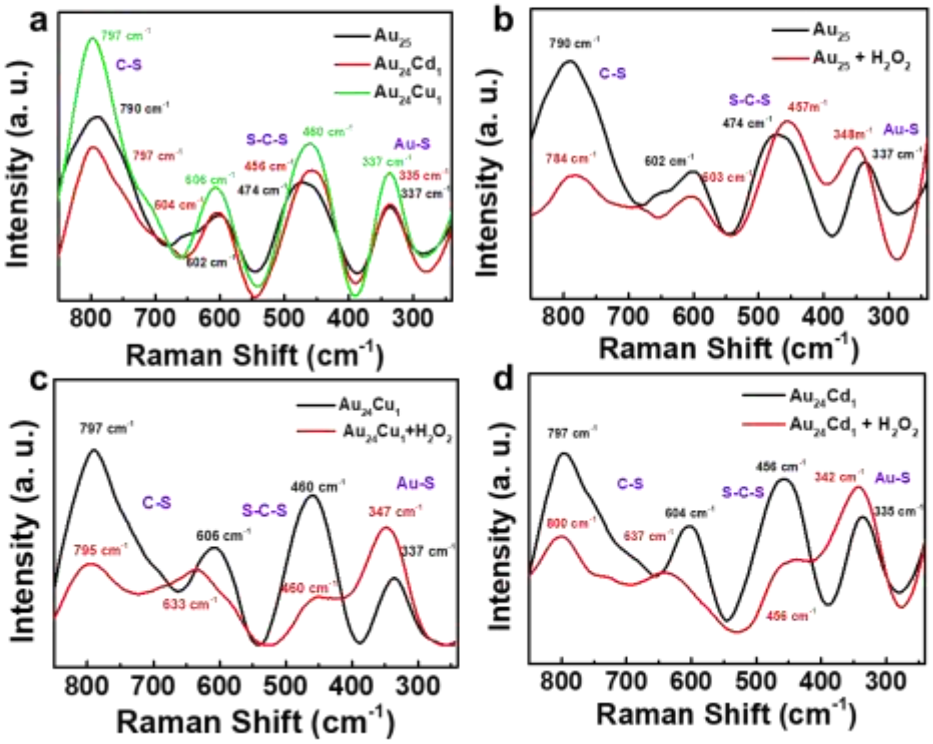

Supplementary Figure 11. a-d Raman spectra of Au_{25}, $Au_{24}Cu_1$ and $Au_{24}Cd_1$ clusterzymes before and after the reaction of H_2O_2. The Raman peaks before and after reaction of H_2O_2 do not change significantly, indicating that the structure are basically unchanged.

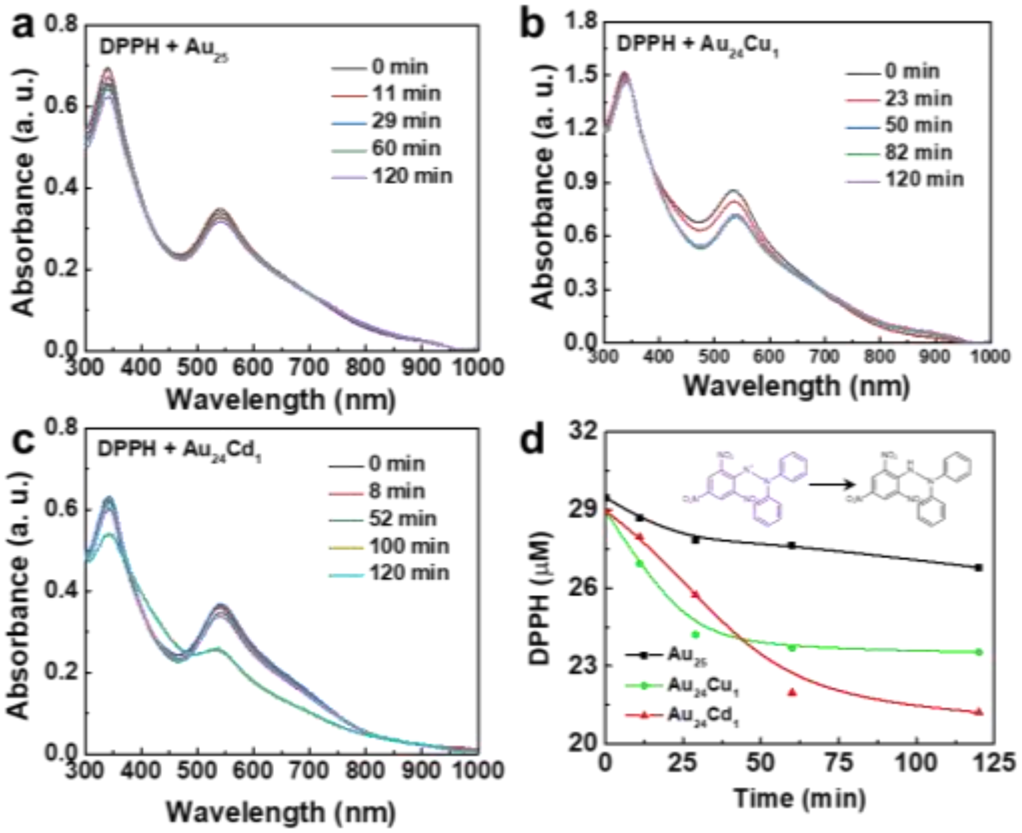

Supplementary Figure 12. Absorption spectra of DPPH• aqueous solution added with 5 ng/μL **a** Au_{25} **b** $Au_{24}Cu_1$ **c** $Au_{24}Cd_1$ from 0 to 120 min. **d** Time-dependent of DPPH• solution treated with or without clusterzymes from the absorbance at 550 nm. It can be seen $Au_{24}Cd_1$ shows the best scavenging effect of DPPH•, while Au_{25} has almost no scavenging effect, indicating that $Au_{24}Cd_1$ has a better scavenging ability for RNS.

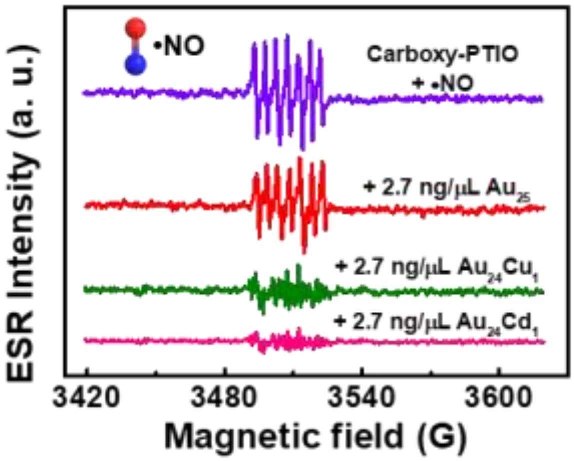

Supplementary Figure 13. •NO scavenging activities of Au_{25}, $Au_{24}Cu_1$ and $Au_{24}Cd_1$ clusterzymes. Using SNAP as the •NO contributor, and Carboxy-PTIO as the capturing agent. It can be seen that $Au_{24}Cd_1$ has the best •NO scavenging activities, while Au_{25} is very poor.

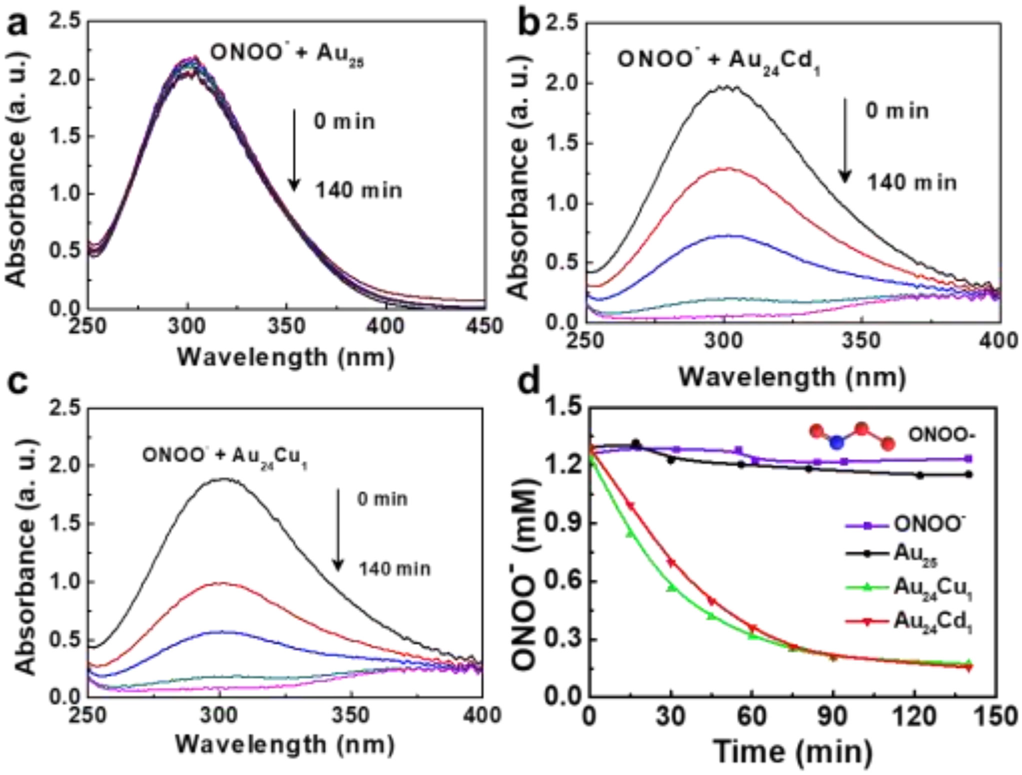

Supplementary Figure 14. Absorption spectra of $ONOO^-$ aqueous solution added with 5 ng/μL **a** Au_{25} **b** $Au_{24}Cd_1$ **c** $Au_{24}Cu_1$ from 0 to 140 min. **d** Time-dependent of $ONOO^-$ solution treated with or without clusterzymes from the absorbance at 302 nm.

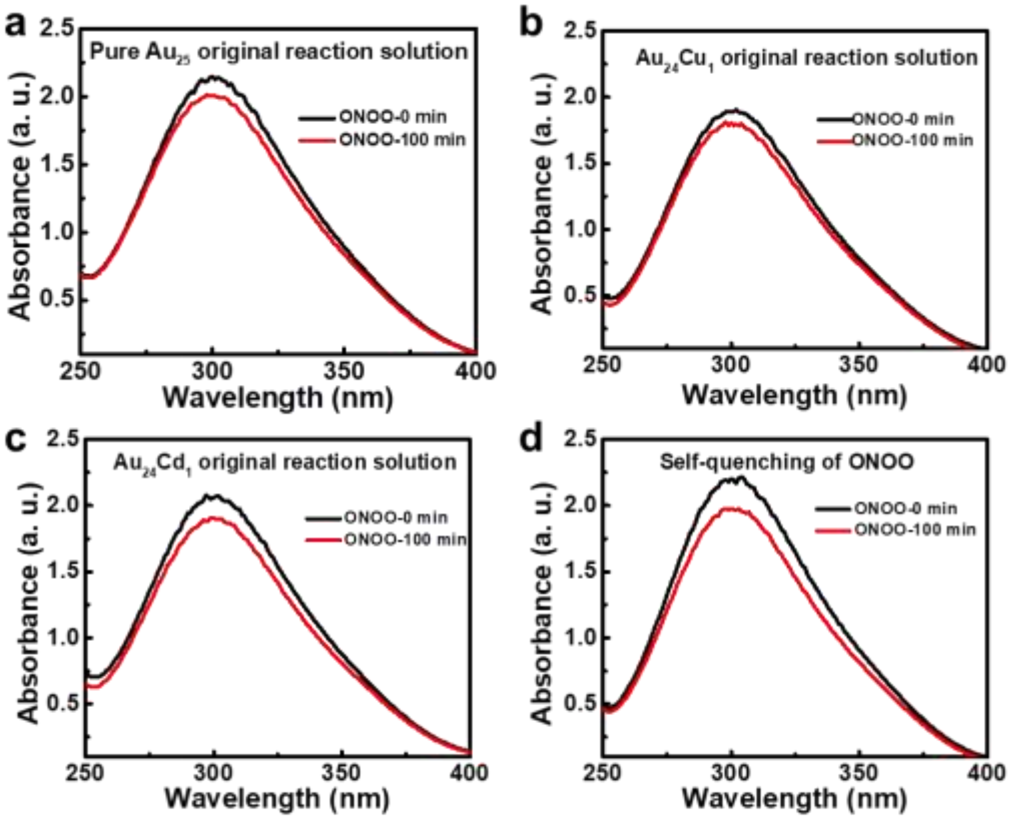

Supplementary Figure 15. a-c Absorption spectra of $ONOO^-$ aqueous solution added with unreacted solution of various clusterzymes (20 μL). **d** Self-quenching of $ONOO^-$ within 100 min. The above results indicate that the $ONOO^-$ quenching has nothing to do with the original reaction solution and self-quenching, and cleaning of $ONOO^-$ is caused by the addition of $Au_{24}Cd_1$ and $Au_{24}Cu_1$.

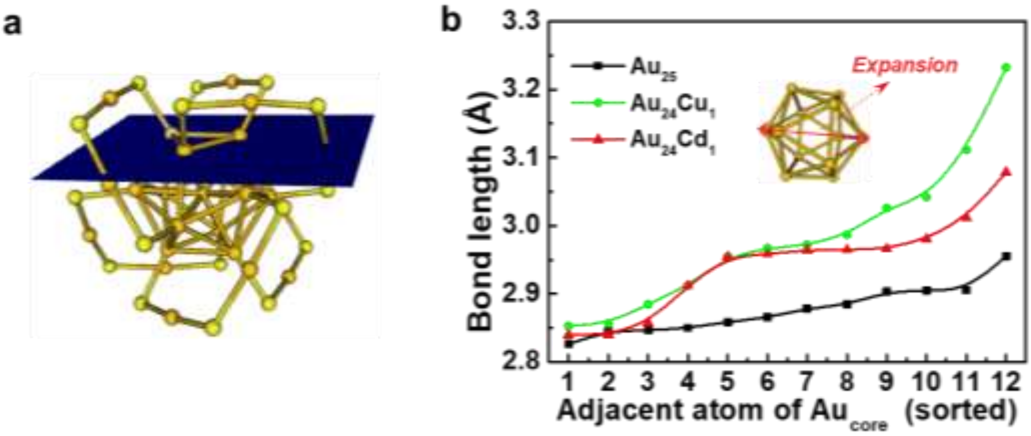

Supplementary Figure 16. a A reaction site exposed to the "bowl" plane. **b** Sorted bond lengths between the central atoms Aucore and their neighboring atoms, indicating the giant lattice expansion of the Au_{13} core.

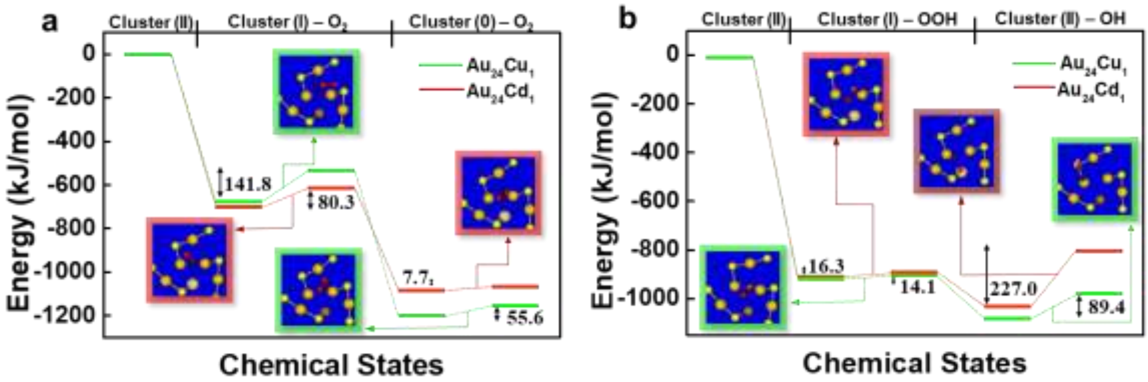

Supplementary Figure 17. Energies profiles and geometry structure of the intermediate states of **a** SOD and **b** CAT process in the lower panel. Small molecules or ions in their transition states are colored by pink.

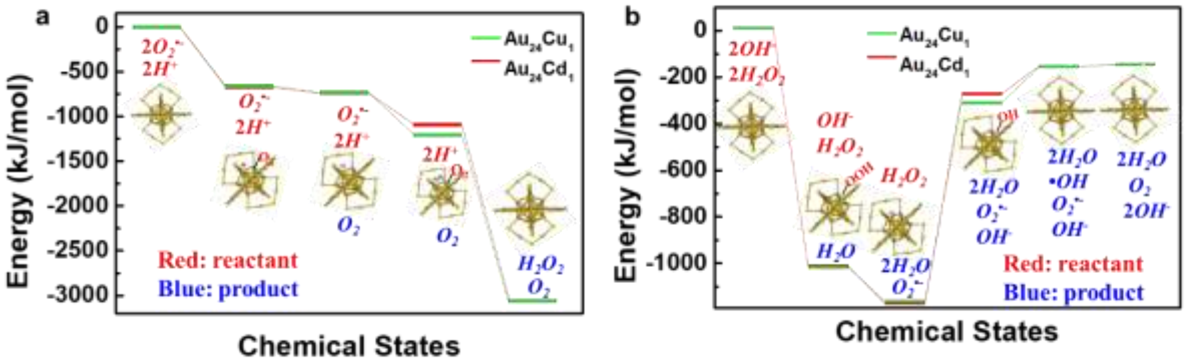

Supplementary Figure 18. Energies profiles of catalytic process of the **a** SOD and **b** CAT processes. The black dotted line indicates that the catalytic products may be connected to other processes.

Complexes	Formula	r(S-X) (Ang)	Eb (kcal/mol)
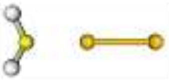	H_2S-Au_2	2.38	-20.26
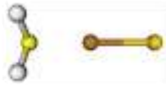	H_2S-CuAu	2.24	-20.16
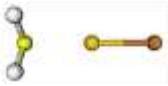	H_2S-CuAu	2.52	-9.11
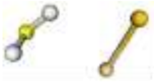	H_2S-CdAu	N. A.	-2.62
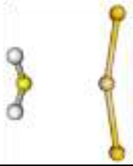	H_2S-$CdAu_2$	3.10	-11.23

Supplementary Figure 19. Investigation of the binding between a H_2S molecule and dimer/trimer of metal atoms. The Cu-Au dimer calculations support the statement, and a S-X bond can be strengthened by extra Au atoms in the S-X-Au_n form but not in the S-Au_n-X form. For the interaction between a Cd atom and a H_2S molecule, our simulation does not produce a valid binding. The interaction between a Cd-Au dimer and a H_2S molecule is also weak. The S-Cd binding shows until we add two Au atoms and the trimer of $CdAu_2$ is formed. The binding is as weak as a trimer of silver atoms, but the Cd appears to be a valid binding point of the S atom. So in this paper, we focus on the surface replacement.

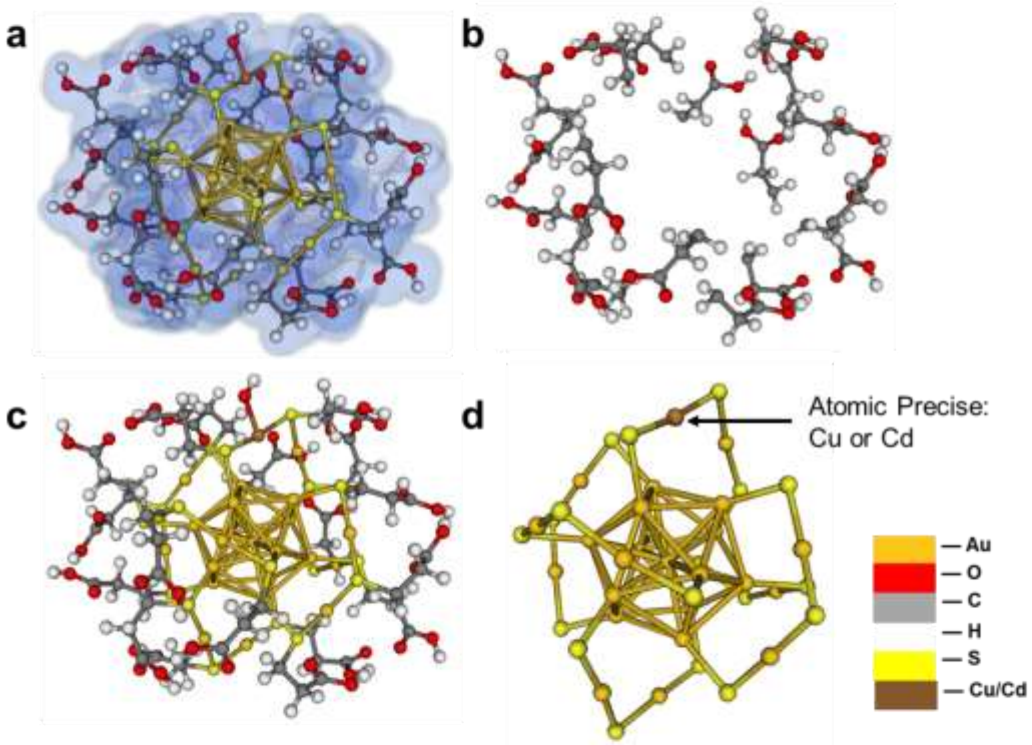

Supplementary Figure 20. a-c Electron cloud distribution and structure of an Au_{25} cluster protected by MPA, and **d** Au_{25} demonstration of Cu or Cd atom substituting: oligomer replacement.

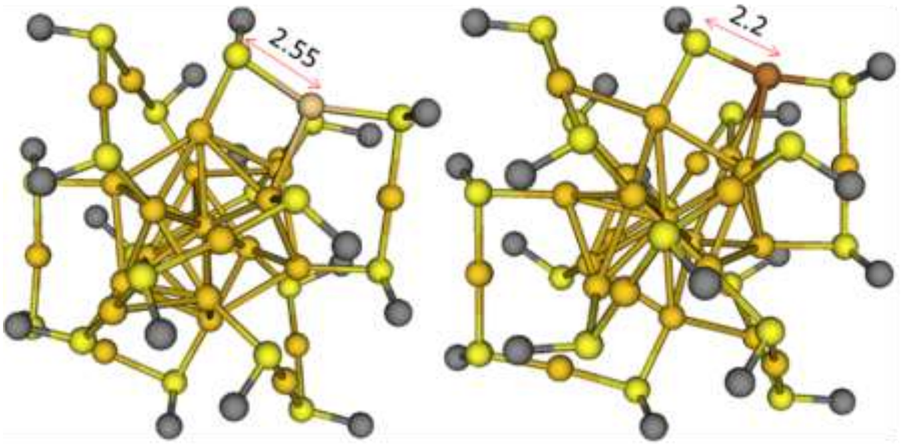

Supplementary Figure 21. S-Cd and S-Cu bonds in optimized structures. Lengths of the bonds are marked above.

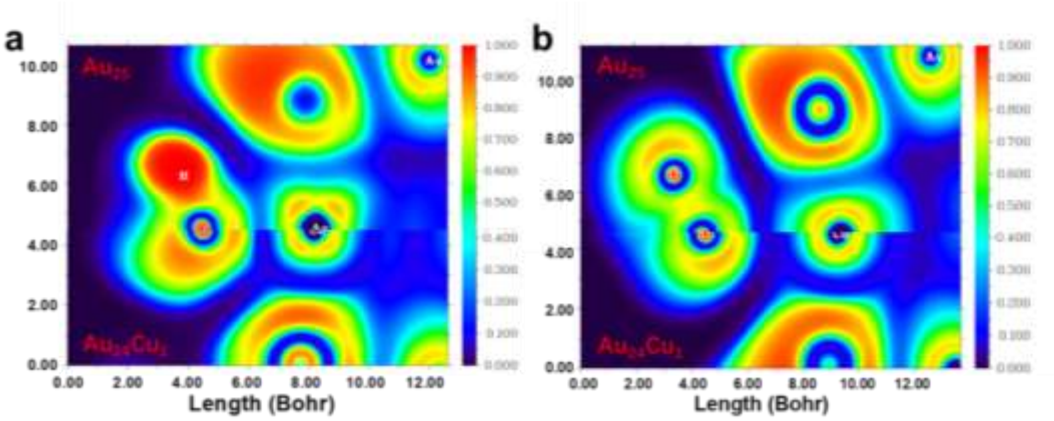

Supplementary Figure 22. a-b The color plot of the electron localization function (elf) of the intermediate structures of the Cu substituted in oligomer site and original Au_{25} clusters. The elf shows the information of bonds and free electrons. the Cu replacement weakens the interactions between the metal and sulfur atoms.

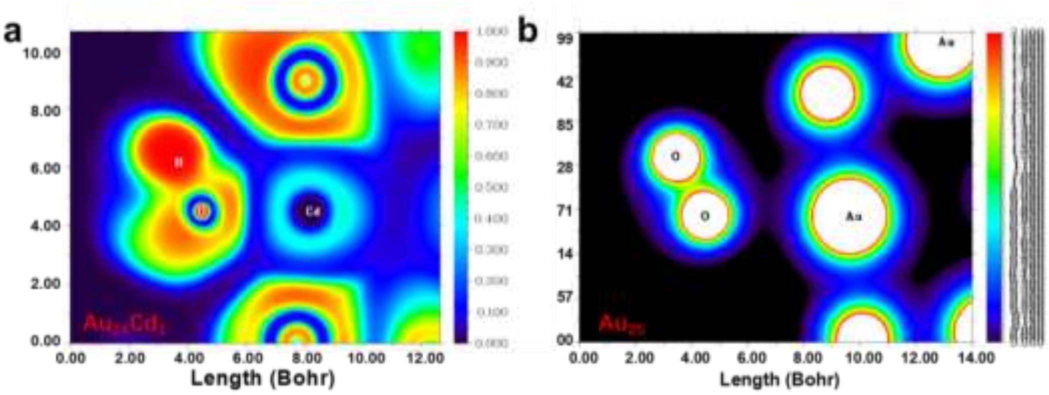

Supplementary Figure 23. a Elf plot and **b** the electrostatic potential (esp) of Au_{25} clusters before and after Cd substituting on oligomer.

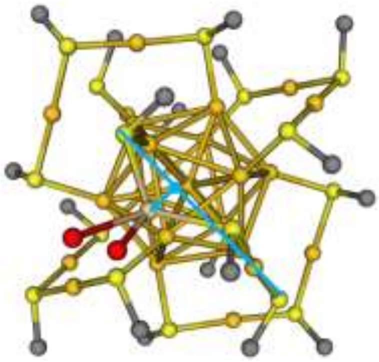

Supplementary Figure 24. An intermediate structure of the angular motion. Blue line: the oligomer plane. Blue arrow: the deviation of Cd atom off the plane.

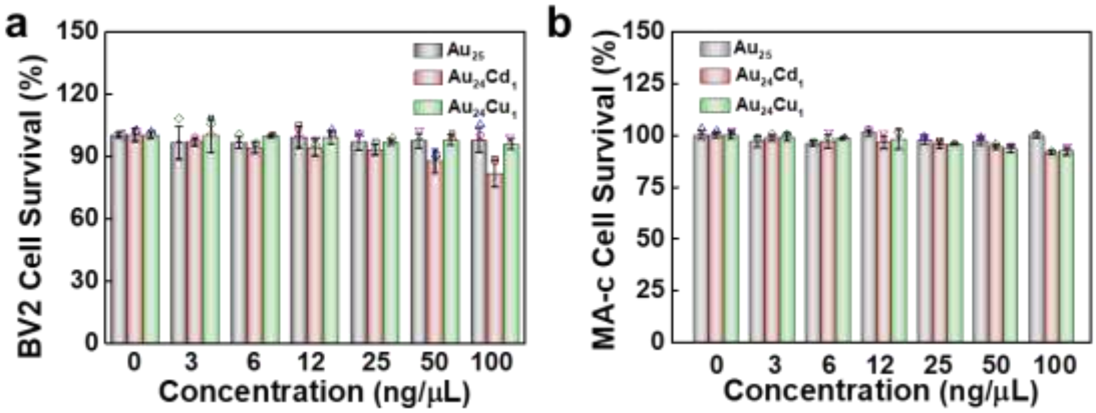

Supplementary Figure 25. Cell viability of **a** BV2 and **b** MA-c cells in the presence of various concentrations of clusterzymes determined by MTT assays (n=5 per group, data are presented as mean ± SD).

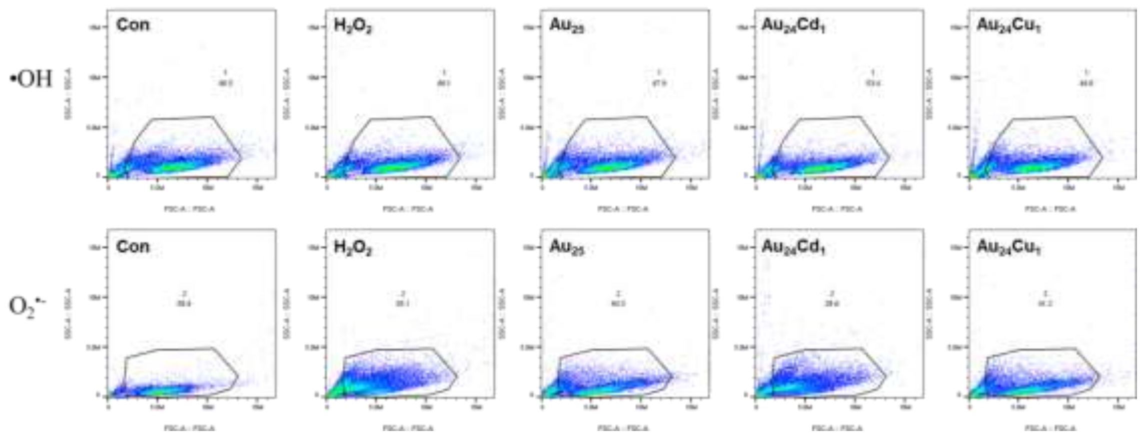

Supplementary Figure 26. Representative gating strategy for analysis about cell staining of •OH (above) and $O_2^{•-}$ (following).

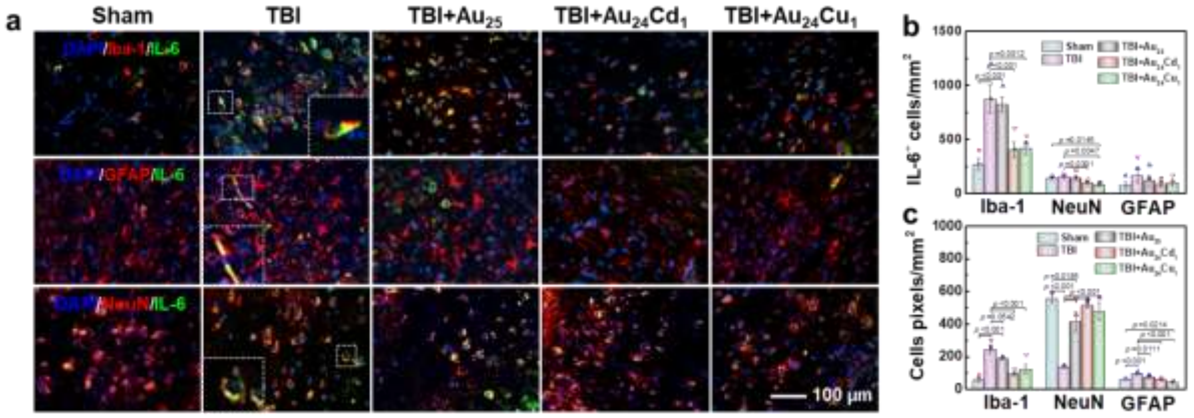

Supplementary Figure 27. a Immunofluorescence co-staining of IL-6 and microglia (Iba-1), astrocytes (GFAP) or neurons (NeuN) in injured cortex 3 days post injury with or without clusterzyme treatment. Quantitative analysis of **b** the number of IL-6^+ expression in different positive cells and **c** the pixels density of Iba-1/NeuN/GFAP cells in injured cortex with or without clusterzymes treatment (n=3 per group). Data are mean ± SEM; Compared with the Sham and TBI group, analyzed by one-way ANOVA with two-sided LSD test (adjusted *p* values are shown). Experiments were repeated independently **a** three times with similar results.

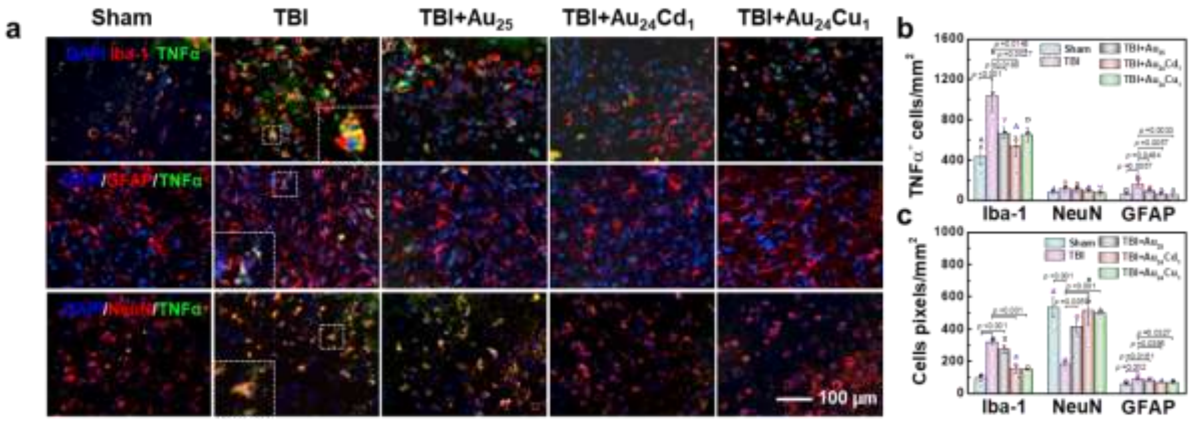

Supplementary Figure 28. a Immunofluorescence co-staining of TNFα and and microglia (Iba-1), astrocytes (GFAP) or neurons (NeuN) in injured cortex at 3 days post-injury with or without clusterzyme treatment. Quantitative analysis of **b** the number of TNFα^{+} expression in different positive cells and **c** the pixels density of Iba-1/NeuN/GFAP cells in injured cortex with or without clusterzymes treatment (n=3 per group). Data are mean ± SEM; Compared with the Sham and TBI group, analyzed by one-way ANOVA with two-sided LSD test (adjusted *p* values are shown). Experiments were repeated independently **a** three times with similar results.

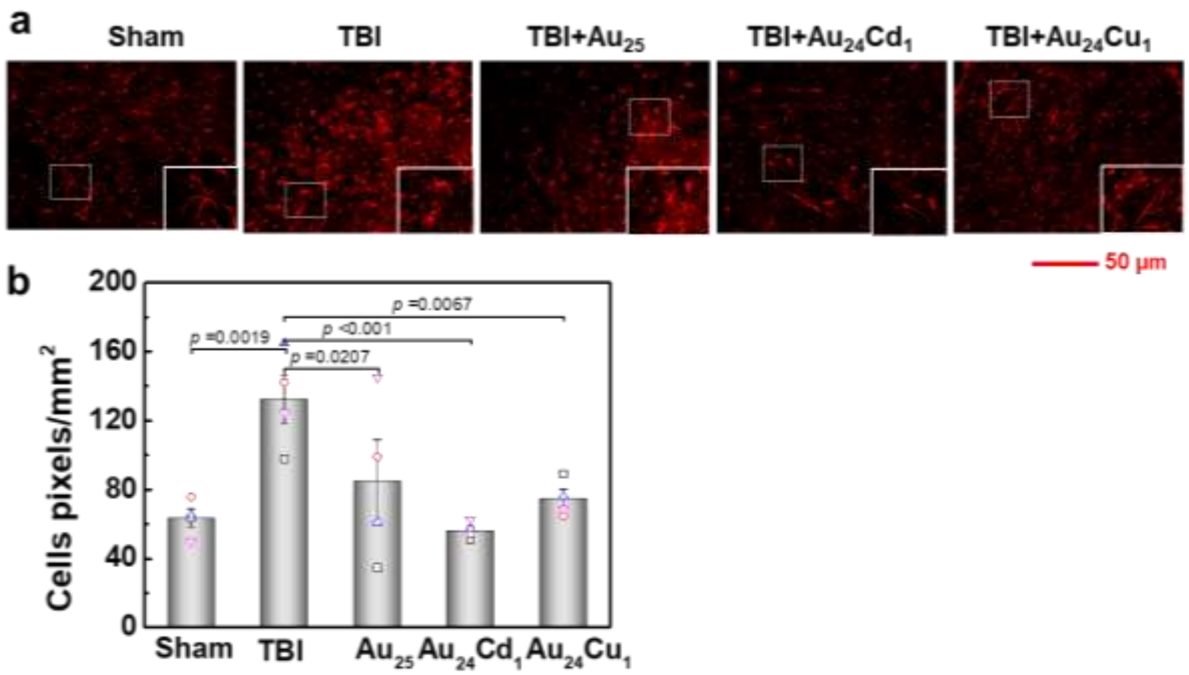

Supplementary Figure 29. a Astrocyte activation levels in the cerebral cortex by GFAP immunofluorescence staining in TBI mice with or without clusterzyme treatment. Insets show a magnified morphology of astrocyte cells. **b** Quantitative analysis of the pixels density of astrocytes (n=3 per group). Data are mean ± SEM; Compared with the Sham and TBI group, analyzed by one-way ANOVA with two-sided LSD test (adjusted p values are shown). Experiments were repeated independently **a** three times with similar results.

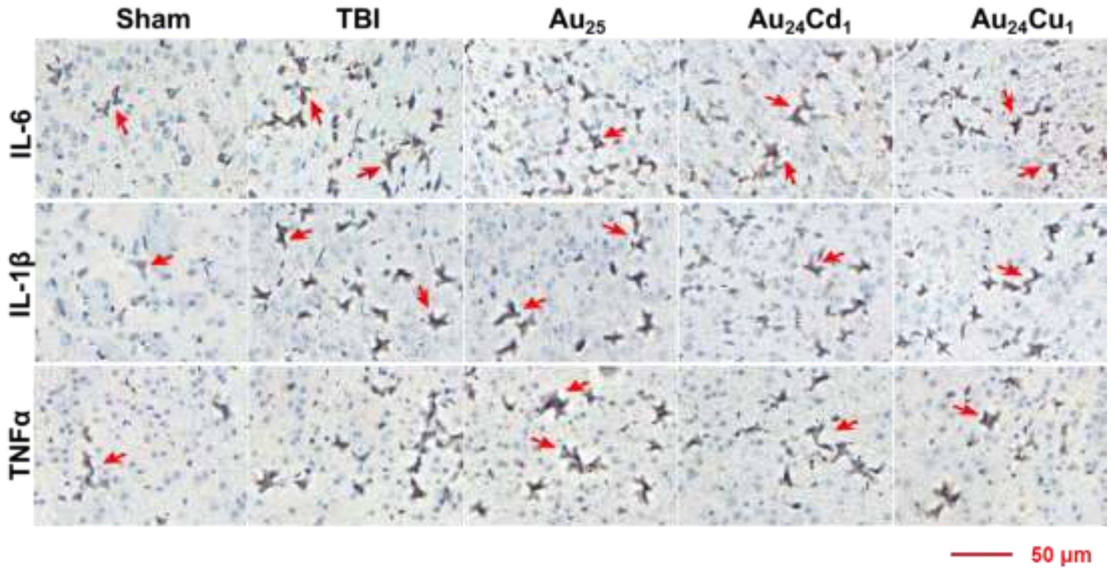

Supplementary Figure 30. Immunohistochemistry of IL-6, IL-1β and TNFα in brain tissues on day 1 post injury (n= 3 per group). Red arrows refer to glial cells that express inflammatory cytokines. Scale bar: 50 μm. Experiments were repeated independently three times with similar results.

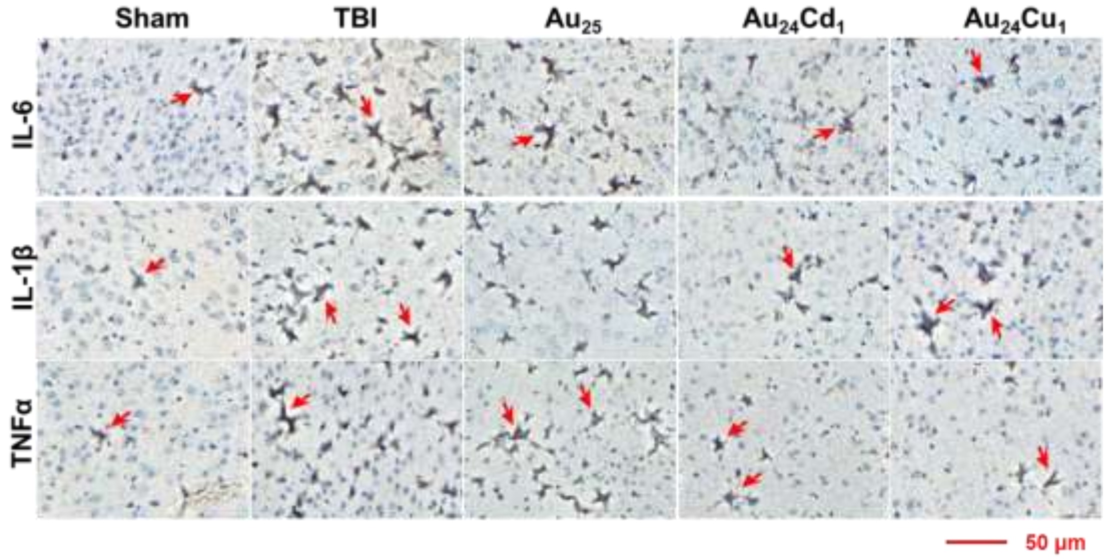

Supplementary Figure 31. Immunohistochemistry of IL-6, IL-1β and TNFα in brain tissues on day 3 post injury (n= 3 per group). Red arrows refer to glial cells that express inflammatory cytokines. Scale bar: 50 μm. Experiments were repeated independently three times with similar results.

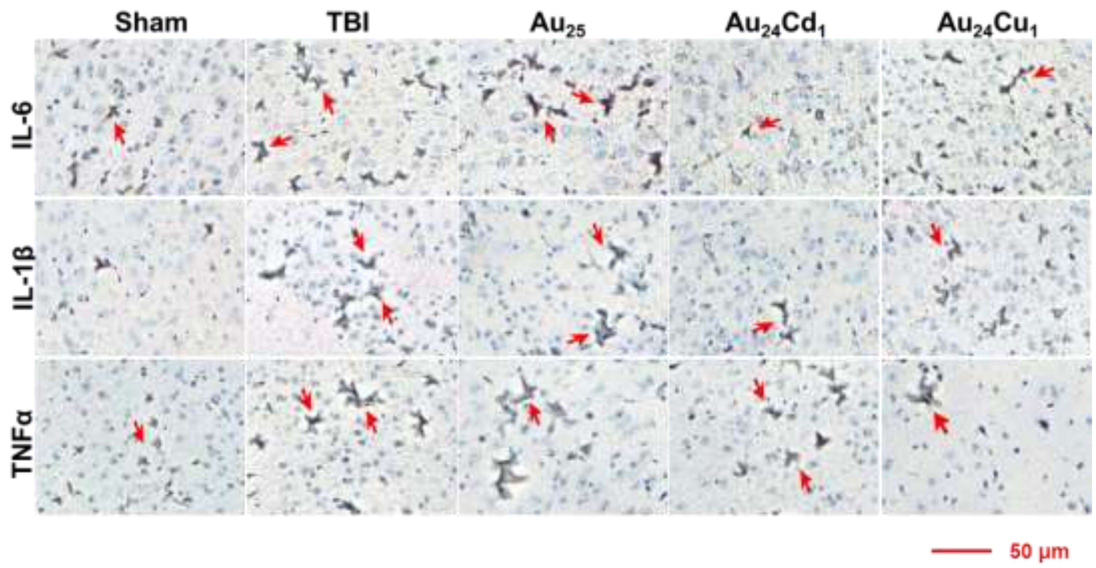

Supplementary Figure 32. Immunohistochemistry of IL-6, IL-1β and TNFα in brain tissues on day 7 post injury (n= 3 per group). Red arrows refer to glial cells that express inflammatory cytokines. Scale bar: 50 μm. Experiments were repeated independently three times with similar results.

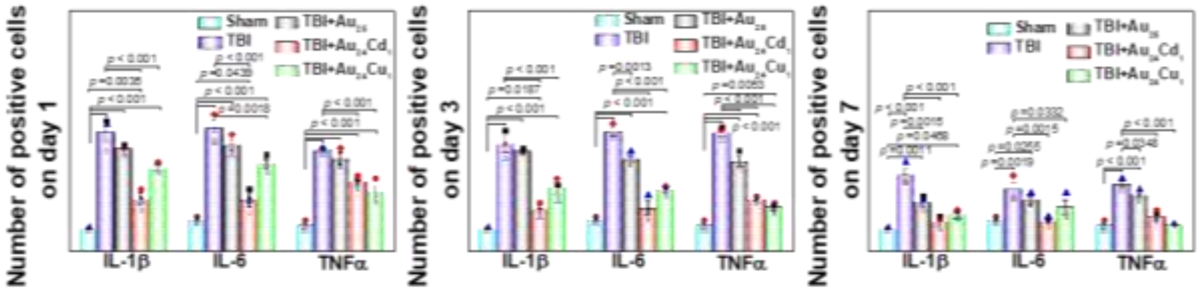

Supplementary Figure 33. Quantitative analysis of immuno-histochemical results including IL-6, IL-1β and TNFα on day 1, 3, and 7 post injury (n=3 per group). Data are mean ± SEM; Compared with the Sham and TBI group, analyzed by one-way ANOVA with two-sided LSD test (adjusted *p* values are shown).

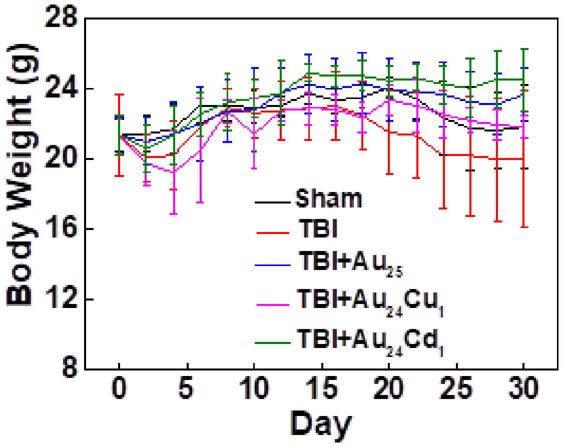

Supplementary Figure 34. Body weights of all TBI mice groups treated with and without clusterzymes changes over time (n=7 per group, data are presented as mean ± SD).

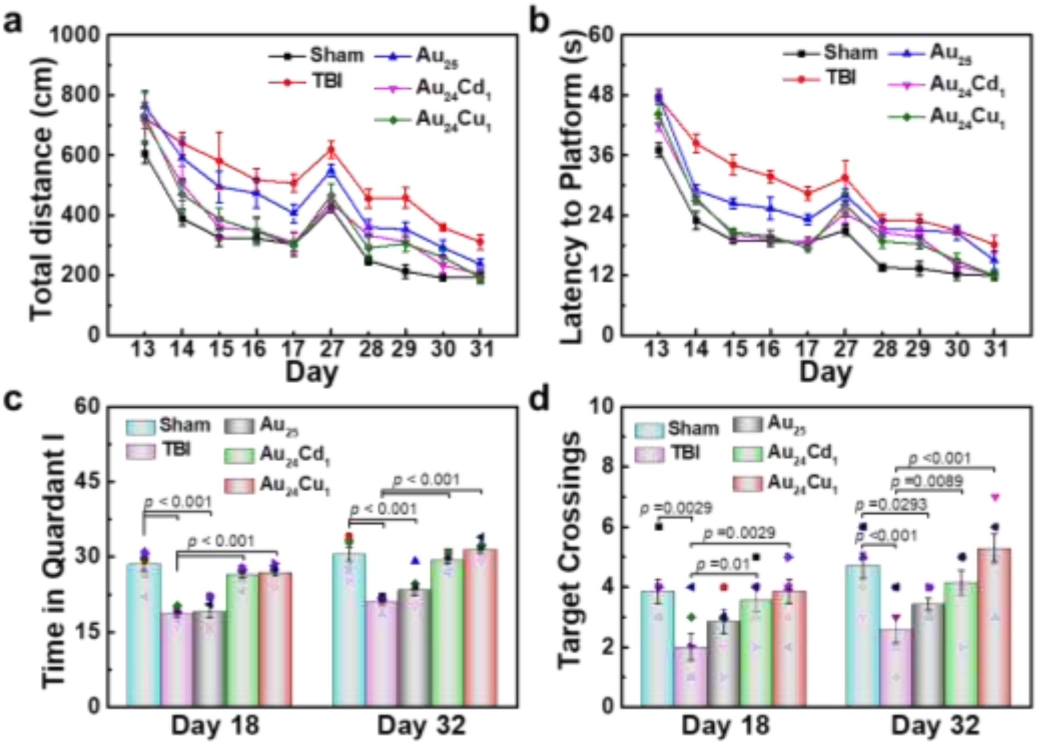

Supplementary Figure 35. Morris water maze tests on days 13-18 and 27-32 in all groups (n=7 per group, data are presented as mean ± SD). **a** Distance traveled (path length) to the hidden platform and **b** latency to locate and rest on the hidden platform recorded for spatial learning trials on days 13-17 and 27-31. **c** Percentage of time during the probe trial spent in the quadrant of tank that previously housed the hidden platform and **d** the number of platform location crossings recorded for the probe trial on day 18 and 32.

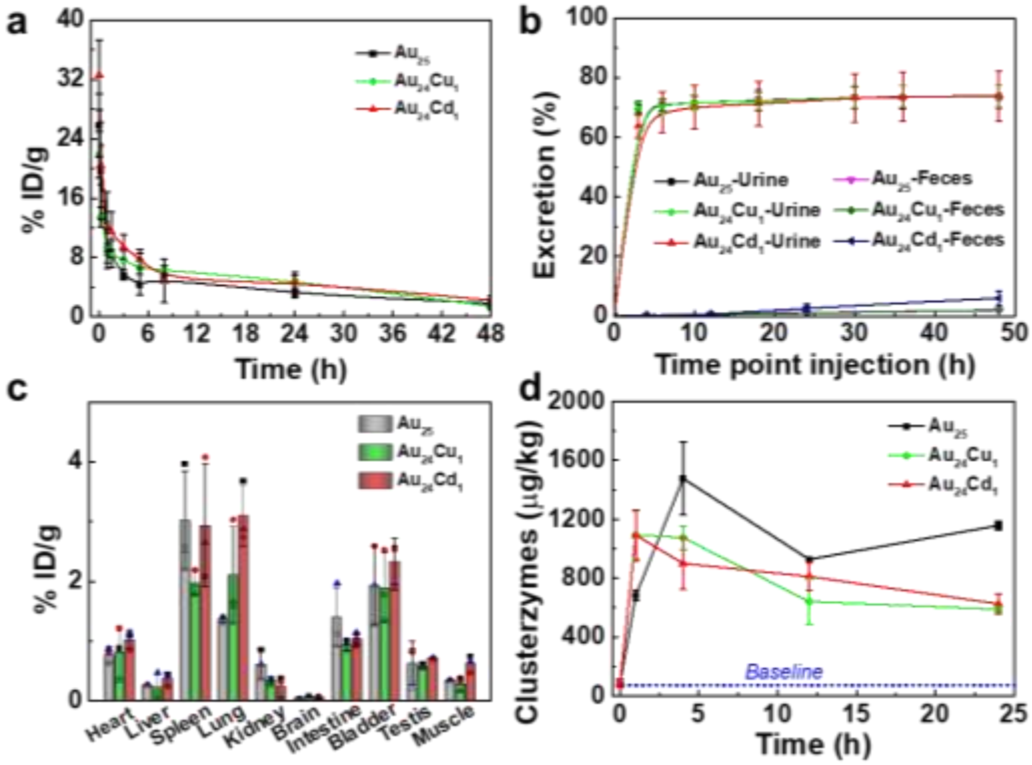

Supplementary Figure 36. a The time activity curves of clusterzymes in blood. **b** Cumulative total excretion including urine and feces of clusterzymes with collected time up to 48 h, showing excellent excretion. **c** Biodistribution of clusterzymes 1 day p.i. (heart, lung, liver, spleen, kidney, muscle, bladder, testicles, intestine and brain, % ID/g = percentage of the injected dose per gram of tissue). **d** Brain uptake and BBB permeability. Injected dose: 5 mg/mL, 200 μL, n=3 per group, data are presented as mean ± SD.

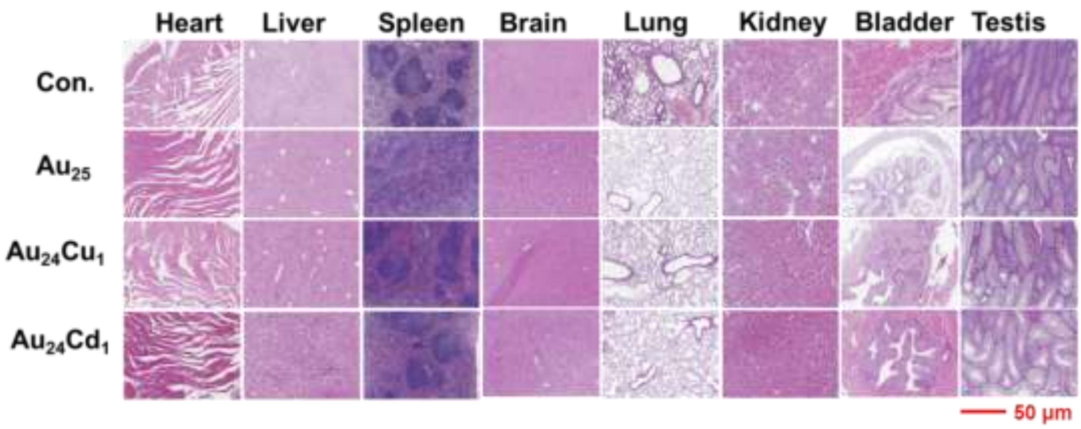

Supplementary Figure 37. Histology of major organs in mice (heart, liver, spleen, lung, kidney, bladder and testis) treated with 200 μL clusterzymes at the concentration of 5 mg/mL after 7 days. Scale bar is 50 μm, n=3 per group. No significant toxic responses were found in all organs. Experiments were repeated independently twice with similar results.

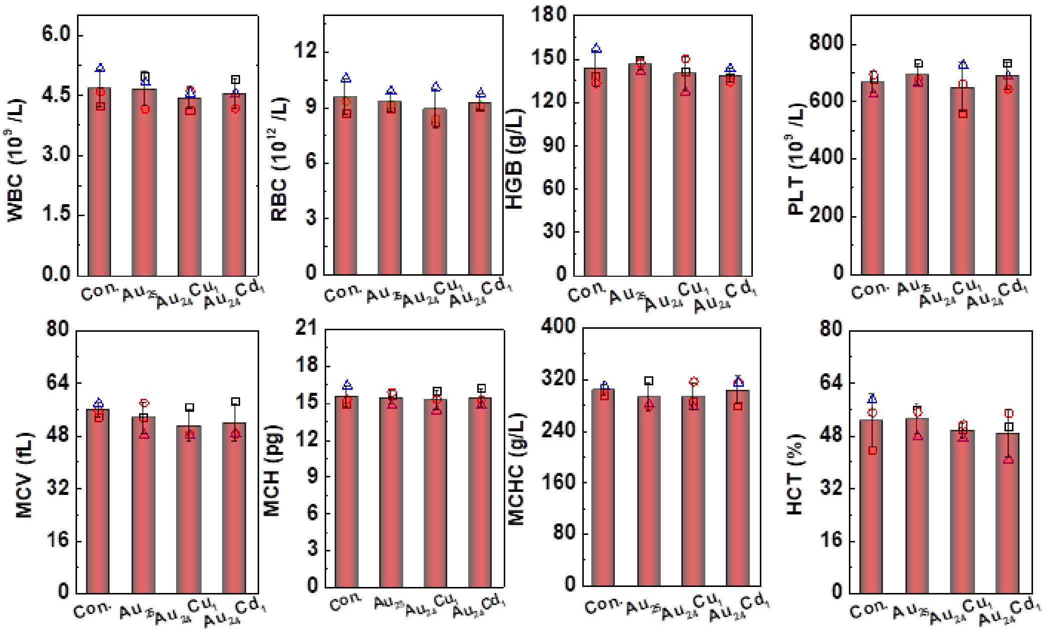

Supplementary Figure 38. Hematology of mice 7 days after being treated with 200 μL clusterzymes at the concentration of 5 mg/mL. The results show the mean and standard error of the mean of white blood cells (WBC), red blood cell (RBC), hematocrit (HCT), mean corpuscular volume (MCV), hemoglobin (HGB), platelets (PLT), mean corpuscular hemoglobin (MCH), and mean corpuscular hemoglobin concentration (MCHC), n=3 per group. Data are presented as mean ± SD.

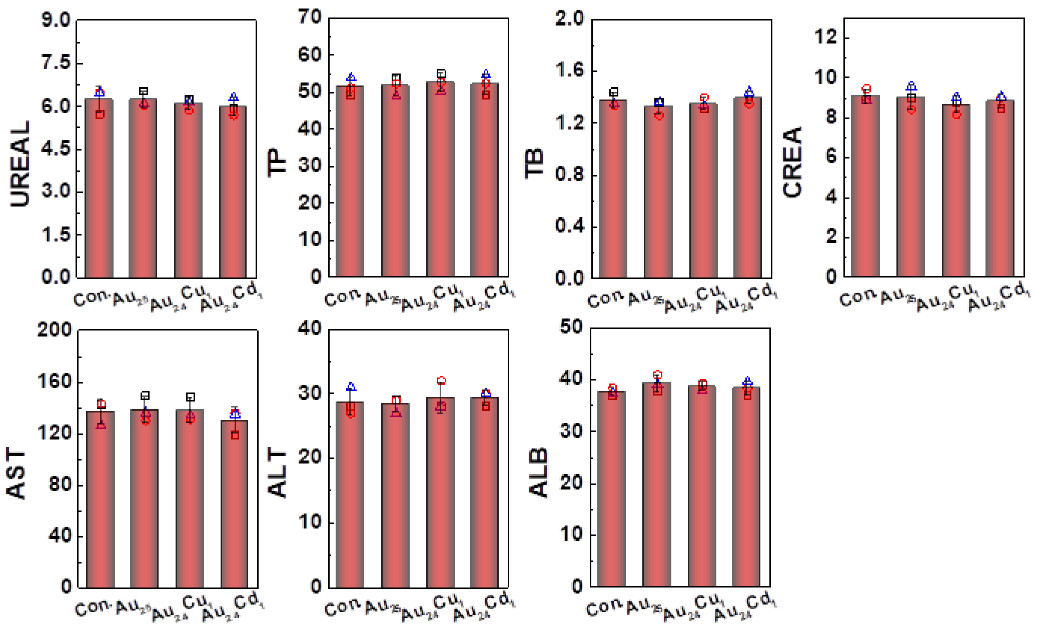

Supplementary Figure 39. Blood biochemistry analysis of mice 7 days after being treated with 200 μL clusterzymes at the concentration of 5 mg/mL. The results show mean and standard error of the mean of ureal, alanine aminotransferase (ALT), albumin (ALB), aspartate aminotransferase (AST), total protein (TP), creatinine (CREA), and total bilirubin (TB), n=3 per group. Data are presented mean ± SD.

Supplementary Table 1. Fitting parameters of Cu K-edge and Cd L_3-edge EXAFS for $Au_{24}Cu_1$ and $Au_{24}Cd_1$. CN is the coordination number, R is the bond length, and σ^2 is the Debye-Waller factor.

$Au_{24}M_1$	CN	R(Å)	$\sigma^2 (10^{-3}$ Å$^2)$	R-factor
Cu-S	1.9±0.2	2.25±0.02	10.8±3.1	0.028
Cd-S	2.3±1.7	2.3±0.42	1.7	0.037

Supplementary Table 2. The CN values in the intermediate structures (including AuCd-OH^{2+}, AuCd-OH^{2+}, AuCd-OH^{2+}, AuCd-OH^{2+}) are generated without the attached units.

Structure	CN (Theoretical)	CN (Experimental)
$AuCd^{2+}$	Cd 3.762	
$AuCu^{2+}$	Cu 2.597	
AuCd-OH^{2+}	Cd 2.501	
AuCu-OH^{2+}	Cu 2.511	
AuCd-OOH^{+}	Cd 2.592	
AuCu-OOH^{+}	Cu 2.081	
Average	Cd 2.952	Cd 2.5
	Cu 2.400	Cu 2.0

Supplementary Table 3. The bond lengths between the ion/segment and the clusterzymes with surface replacement in the optimized intermediate structures in CAT processes. the O-O bonds are also provided.

	Doped atom	Nearest gold atom	O-O bond length
Cu (I) OOH	2.09	2.12	1.49
Cu (I) OOH ts	1.97	2.09	1.49
Cu (I) OH	5.01	3.16	
Cu (I) OH ts	3.57	2.27	
Cd (I) OOH	2.23	2.32	1.50
Cd (I) OOH ts	2.37	2.21	1.44
Cd (I) OH	2.92	3.23	
Cd (I) OH ts	2.22	2.30	

Supplementary Table 4. The bond lengths between the ion/segment and the clusterzymes with surface replacement in the optimized intermediate structures in SOD processes. the O-O bonds are also provided.

	Doped atom	Nearest gold atom	O-O bond length
Cu (I)	3.55	2.76	1.24
Cu (I) ts	2.77	3.14	1.24
Cu (0)	3.36	3.26	1.40
Cu (0) ts	3.17	3.32	1.24
Cd (I)	2.78	3.09	1.24
Cd (I) ts	2.75	3.28	1.24
Cd (0)	3.13	3.38	1.24
Cd (0) ts	3.02	3.32	1.24

Supplementary Table 5. The bond lengths between the ion/segment and the clusterzymes with oligomer replacement in the optimized intermediate structures in SOD processes. the O-O bonds are also provided.

SOD	O-X distance	S-X bond length	O-O bond length
Cu (I)	3.69	2.25	1.25
Cu (I) ts	2.08	2.27	1.31
Cu (0)	3.98	2.25	1.26
Cu (0) ts	3.43	2.25	1.25
Cd (I)	3.32	2.56	1.26
Cd (I) ts	3.45	2.56	1.26
Cd (0)	2.26	2.57; 2.60	1.39
Cd (0) ts	2.27	2.63; 2.85	1.35
Superoxide	-	-	1.41

Supplementary Table 6. The bond lengths between the ion/segment and the clusterzymes with oligomer replacement in the optimized intermediate structures in CAT processes. the O-O bonds are also provided.

CAT	O-X distance	S-X bond length	O-O bond length
Cu (I)	2.02	2.28	1.46
Cu (I) ts	2.07	2.27	1.45
Cu (II)	2.07	2.27	-
Cu (II) ts	2.04	2.25	-
Cd (I)	2.15	2.60; 2.65	1.58
Cd (I) ts	2.13	2.89; 2.62	1.60
Cd (II)	2.08	2.67; 2.60	-
Cd (II) ts	2.04	2.60; 2.70	-
