# Supplementary material for: Catalytically potent and selective clusterzymes for modulation of neuroinflammation through single-atom substitutions
Source: Nat Commun. 2021 Jan 7;12:114. doi: 10.1038/s41467-020-20275-0 (PMC7791071; doi:10.1038/s41467-020-20275-0)
Supplement: Supplementary file 3 — Reporting Summary [file 41467_2020_20275_MOESM3_ESM.pdf]

nature research

Corresponding author(s): Xiaodong Zhang

Last updated by author(s): Nov 5, 2020

Reporting Summary

Nature Research wishes to improve the reproducibility of the work that we publish. This form provides structure for consistency and transparency in reporting. For further information on Nature Research policies, see our Editorial Policies and the Editorial Policy Checklist.

Statistics

For all statistical analyses, confirm that the following items are present in the figure legend, table legend, main text, or Methods section.

n/a	Confirmed	
☐	☒	The exact sample size (n) for each experimental group/condition, given as a discrete number and unit of measurement
☐	☒	A statement on whether measurements were taken from distinct samples or whether the same sample was measured repeatedly
☐	☒	The statistical test(s) used AND whether they are one- or two-sided<br>*Only common tests should be described solely by name; describe more complex techniques in the Methods section.*
☒	☐	A description of all covariates tested
☐	☒	A description of any assumptions or corrections, such as tests of normality and adjustment for multiple comparisons
☐	☒	A full description of the statistical parameters including central tendency (e.g. means) or other basic estimates (e.g. regression coefficient) AND variation (e.g. standard deviation) or associated estimates of uncertainty (e.g. confidence intervals)
☐	☒	For null hypothesis testing, the test statistic (e.g. F, t, r) with confidence intervals, effect sizes, degrees of freedom and P value noted<br>*Give P values as exact values whenever suitable.*
☒	☐	For Bayesian analysis, information on the choice of priors and Markov chain Monte Carlo settings
☒	☐	For hierarchical and complex designs, identification of the appropriate level for tests and full reporting of outcomes
☒	☐	Estimates of effect sizes (e.g. Cohen's d, Pearson's r), indicating how they were calculated

Our web collection on statistics for biologists contains articles on many of the points above.

Software and code

Policy information about availability of computer code

Data collection	BD AccuriTM C6
Data analysis	In this study, Microsoft Excel 2010, Origin 9.0, ARTEMIS, IFEFFIT, XPSPEAK41, Berny algorithm, SPSS 19, ImageJ, Flowjo 10.6.2 were used to analyze the data.

For manuscripts utilizing custom algorithms or software that are central to the research but not yet described in published literature, software must be made available to editors and reviewers. We strongly encourage code deposition in a community repository (e.g. GitHub). See the Nature Research guidelines for submitting code & software for further information.

Data

Policy information about availability of data

All manuscripts must include a data availability statement. This statement should provide the following information, where applicable:
- Accession codes, unique identifiers, or web links for publicly available datasets
- A list of figures that have associated raw data
- A description of any restrictions on data availability

The data that support the findings of this study are available from the corresponding author upon reasonable request. Source data are provided with this paper.

Field-specific reporting

Life sciences study design

All studies must disclose on these points even when the disclosure is negative.

Sample size	No sample-size calculation was performed. All biologically based assays were performed with the usual and sufficient sample size setting determined by previous experiments. These sample sizes were sufficient for a statistical analysis. All experiments reported have n number and repetitions reported.
Data exclusions	No data was excluded from the analysis.
Replication	Results shown in the manuscript are representative of at least two similar experiments. All our attempts at replication were successful.
Randomization	Our samples/organisms were allocated randomly.
Blinding	In all experiments, investigators were blinded to group allocation during data collection and processing.

Reporting for specific materials, systems and methods

We require information from authors about some types of materials, experimental systems and methods used in many studies. Here, indicate whether each material, system or method listed is relevant to your study. If you are not sure if a list item applies to your research, read the appropriate section before selecting a response.

Materials & experimental systems

n/a	Involved in the study	
☐	☒	Antibodies
☐	☒	Eukaryotic cell lines
☒	☐	Palaeontology and archaeology
☐	☒	Animals and other organisms
☒	☐	Human research participants
☒	☐	Clinical data
☐	☒	Dual use research of concern

Methods

n/a	Involved in the study	
☒	☐	ChIP-seq
☐	☒	Flow cytometry
☒	☐	MRI-based neuroimaging

Antibodies

Antibodies used

Antibodies used:
Anti-TNFα antibody, Abcam, ab183218. Lot: GR284782-19, source: rabbit.
Anti-IL6 antibody Bioss, bs-0782R.
Anti-IL 1β Bioss, bs-0812R.
Antibody Anti-NeuN antibody, GeneTex, GTX00837. Lot: 822003252, source: chicken.
Anti-Iba1 antibody, Abcam, ab48004. Lot: GR123692-54, source: goat.
Anti-GFAP, Abcam, ab90601. Lot: GR3347908-1, source: sheep.
CoraLite488-conjugated Affinipure Donkey Anti-Rabbit IgG (H+L), Proteintech, SA00013-6. Lot: 20000227.
Goat Anti-Chicken IgY H&L (Alexa Fluor 647), Abcam, ab150171. Lot: GR3312420-1.
Donkey Anti-Sheep IgG H&L (Alexa Fluor 647), Abcam, ab150179. Lot: GR3285522-2.
Donkey Anti-Goat IgG H&L (Alexa Fluor 647), Abcam, ab150131. Lot: GR3246238-4, source:donkey.
Anti-TNFα antibody, Abbkine, ABP0127. Lot: ATTAU1401, source:rabbit.
Anti-IL-6 antibody, Proteintech, 66146-1-lg. Lot: 10010760, source:mouse.
Anti-IL 1β, Abbkine, ABP52932. Lot: ATTAU1401, source:rabbit.
Anti-TNFα antibody, Abcam, ab34674. Lot: GR135625-52.
Anti-IL-6 antibody, Abcam, ab7737. Lot: GR3242890-10.
Anti-IL-1β antibody, Abcam, ab234437. Lot: GR3315061-2.
β-actin antibody, Sigma-Aldrich, A5441.

Validation

All the antibodies commercially purchased from these companies: link provided :1) https://www.abcam.cn/, 2) http://www.ptgcn.com/, 3) https://www.genetex.cn/, 4) https://www.biossusa.com/, 5) https://www.abbkine.com/, 6) https://www.sigmaaldrich.com/. Each of the antibody can be searched by their clone numbers on their respective website. In general all of the antibodies were quality control tested by immunofluorescent staining with flow cytometric analysis by the companies.
Anti-TNFα antibody, Abcam, ab183218, provided data in manuscript: IF.
Anti-IL-6 antibody Bioss, bs-0782R, provided data in manuscript: IF.
Anti-IL-1β, Bioss, bs-0812R, provided data in manuscript: IF.
Antibody anti-NeuN antibody, GeneTex, GTX00837, provided data in manuscript:IF.
Anti-Iba1 antibody, Abcam, ab48004, provided data in manuscript: IF.
Anti-GFAP, Abcam, ab90601, provided data in manuscript: IF.
CoraLite488-conjugated Affinipure Donkey Anti-Rabbit IgG (H+L), Proteintech, SA00013-6, provided data in manuscript: IF.

Goat Anti-Chicken IgY H&L (Alexa Fluor 647), Abcam, ab150171, provided data in manuscript: IF.<br>Donkey Anti-Sheep IgG H&L (Alexa Fluor 647), Abcam, ab150179, provided data in manuscript: IF.<br>Donkey Anti-Goat IgG H&L (Alexa Fluor 647), Abcam, ab150131, provided data in manuscript:IF.<br>Anti-TNFα, antibody, Abbkine, ABP0127, provided data in manuscript: IHC.<br>Anti-IL-6, antibody, Proteintech, 66146-1-lg, provided data in manuscript: IHC.<br>Anti-IL-1β, Abbkine, ABP52932, provided data in manuscript: IHC.<br>Anti-TNFα antibody, Abcam, ab34674, provided data in manuscript: WB.<br>Anti-IL-6 antibody, Abcam, ab7737, provided data in manuscript:WB.<br>Anti-IL-1β antibody, Abcam, ab234437, provided data in manuscript:WB.<br>β-actin antibody, Sigma-Aldrich, A5441, provided data in manuscript:WB.

Eukaryotic cell lines

Policy information about cell lines

Cell line source(s)	HT22 cells were obtained from the Institute of Radiation Medicine, Chinese Academy of Medical Sciences and Peking Union Medical College. BV2 cells, MA-c cells were obtained from Tianjin Huanhu Hospital.
Authentication	None of the cell lines were authenticated.
Mycoplasma contamination	Cell line were not contaminated by mycoplasma
Commonly misidentified lines (See ICLAC register)	No commonly misidentified cell line was used.

Animals and other organisms

Policy information about studies involving animals; ARRIVE guidelines recommended for reporting animal research

Laboratory animals	C57BL/6J mice, male, 7-9 weeks.
Wild animals	The study did not involve wild animals.
Field-collected samples	No field-collected samples were used in this study.
Ethics oversight	The ethical oversight is subject to the approval and guidance of the Institute of Radiation Medicine, Chinese Academy of Medical Sciences.

Note that full information on the approval of the study protocol must also be provided in the manuscript.

Dual use research of concern

Policy information about dual use research of concern

Hazards

Could the accidental, deliberate or reckless misuse of agents or technologies generated in the work, or the application of information presented in the manuscript, pose a threat to:

No	Yes	
☒	☐	Public health
☒	☐	National security
☒	☐	Crops and/or livestock
☒	☐	Ecosystems
☒	☐	Any other significant area

Experiments of concern

Does the work involve any of these experiments of concern:

No	Yes	
☒	☐	Demonstrate how to render a vaccine ineffective
☒	☐	Confer resistance to therapeutically useful antibiotics or antiviral agents
☒	☐	Enhance the virulence of a pathogen or render a nonpathogen virulent
☒	☐	Increase transmissibility of a pathogen
☒	☐	Alter the host range of a pathogen
☒	☐	Enable evasion of diagnostic/detection modalities
☒	☐	Enable the weaponization of a biological agent or toxin
☒	☐	Any other potentially harmful combination of experiments and agents

Flow Cytometry

Plots

Confirm that:

☒ The axis labels state the marker and fluorochrome used (e.g. CD4-FITC).

☒ The axis scales are clearly visible. Include numbers along axes only for bottom left plot of group (a 'group' is an analysis of identical markers).

☒ All plots are contour plots with outliers or pseudocolor plots.

☒ A numerical value for number of cells or percentage (with statistics) is provided.

Methodology

Sample preparation	HT22 cells were treated for 6 hours under 100 μM H2O2 conditions, and were incubated with clusterzymes for another 18 hours. Then cells were incubated with fluorescence probes. The quantitative analysis of free radical was conducted by a FACS flow cytometer (BD AccuriTM C6).
Instrument	A FACS flow cytometer (BD AccuriTM C6)
Software	BD AccuriTM C6 and Flowjo
Cell population abundance	1×106 cells/mL
Gating strategy	Cells were gated based on size and granularity by forward and side scatter (SSC-A versus FCS-A). Then, cell gate is analyzed for fluorescence intensity to determine the scavenging ability of clusterzymes. Among samples, control is recognized as the negative group, and H2O2 is referred as the positive group.

☒ Tick this box to confirm that a figure exemplifying the gating strategy is provided in the Supplementary Information.
